# Improving Coverage and Compliance in Mass Drug Administration for the Elimination of LF in Two ‘Endgame’ Districts in Indonesia Using Micronarrative Surveys

**DOI:** 10.1371/journal.pntd.0005027

**Published:** 2016-11-03

**Authors:** Alison Krentel, Rita Damayanti, Christiana Rialine Titaley, Nugroho Suharno, Mark Bradley, Timothy Lynam

**Affiliations:** 1 Department of Infectious and Tropical Diseases, London School of Hygiene and Tropical Medicine, London, United Kingdom; 2 Center for Health Research, Faculty of Public Health, Universitas Indonesia, Jakarta, Indonesia; 3 Global Health Programs, GlaxoSmithKline, London, United Kingdom; 4 Reflecting Society Pty Ltd, Townsville, Australia; Erasmus MC, NETHERLANDS

## Abstract

**Background:**

As the Global Programme to Eliminate Lymphatic Filariasis (LF) approaches its 2020 goal, an increasing number of districts will enter the endgame phase where drug coverage rates from mass drug administration (MDA) are used to assess whether MDA can be stopped. As reported, the gap between reported and actual drug coverage in some contexts has overestimated the true rates, thus causing premature administration of transmission assessment surveys (TAS) that detect ongoing LF transmission. In these cases, districts must continue with additional rounds of MDA. Two districts in Indonesia (Agam District, Depok City) fit this criteria—one had not met the pre-TAS criteria and the other, had not passed the TAS criteria. In both cases, the district health teams needed insight into their drug delivery programs in order to improve drug coverage in the subsequent MDA rounds.

**Methodology/Principal Findings:**

To inform the subsequent MDA round, a micronarrative survey tool was developed to capture community members’ experience with MDA and the social realm where drug delivery and compliance occur. A baseline survey was implemented after the 2013 MDA in endemic communities in both districts using the EPI sampling criteria (n = 806). Compliance in the last MDA was associated with perceived importance of the LF drugs for health (p<0.001); perceived safety of the LF drugs (p<0.001) and knowing someone in the household has complied (p<0.001). Results indicated that specialized messages were needed to reach women and younger men. Both districts used these recommendations to implement changes to their MDA without additional financial support. An endline survey was performed after the 2014 MDA using the same sampling criteria (n = 811). Reported compliance in the last MDA improved in both districts from 57% to 77% (p<0.05). Those who reported having ever taken the LF drug rose from 79% to 90% (p<0.001) in both sites.

**Conclusions/Significance:**

Micronarrative surveys were shown to be a valid and effective tool to detect operational issues within MDA programs. District health staff felt ownership of the results, implementing feasible changes to their programs that resulted in significant improvements to coverage and compliance in the subsequent MDA. This kind of implementation research using a micronarrative survey tool could benefit underperforming MDA programs as well as other disease control programs where a deeper understanding is needed to improve healthcare delivery.

## Author Summary

This research describes the process used to assist two districts endemic for lymphatic filariasis (LF) in Indonesia to better understand the reasons why their LF elimination programs have had suboptimal results. A novel survey design was used to collect stories about people’s direct experiences with mass drug administration (MDA) for LF. These questionnaires also explored the reasons community members took or did not take the LF drugs. Following MDA in 2013, two baseline surveys in endemic communities provided insight into the district MDA programs. Together with district health officials, feasible recommendations were provided before the next MDA round in 2014. Uptake of these recommendations by the districts was high, although no additional funding was made available for programmatic changes. As a result, both districts reported significant improvements in their MDA coverage and compliance rates after the endline surveys were completed in 2015. This demonstrated the utility of the survey tool and process to impact change and improvement in MDA programs.

## Introduction

More than fifteen years ago, the Global Programme to Eliminate Lympatic Filariasis (GPELF) was launched with the goal to interrupt transmission of the disease in endemic countries by 2020 [1]. Considerable progress in reducing transmission and burden of disease has been made since World Health Assembly Resolution 50.29 prioritized the elimination of lymphatic filariasis (LF) in 1997. Since the start of LF elimination, there has been an estimated 46% reduction of the population living at risk for LF infection [2], over 96 million LF cases cured or prevented [3, 4] as well as billions of dollars of direct economic benefits in endemic countries [5].

At the end of 2014, of the 73 countries known to be endemic for lymphatic filariasis (LF), 55 required ongoing mass drug administration (MDA) as the recommended preventive chemotherapy (PC) to eliminate LF [4]. Eleven endemic countries still need to begin MDA and 23 countries have less than 100% geographical coverage [4]. As 2020 approaches, there is an increased urgency to scale up activities in these remaining countries. On the other side of the spectrum, implementation units (IUs) that have completed at least five effective MDA rounds qualify for Transmission Assessment Surveys (TAS) to evaluate the level of LF transmission in the population and to determine if MDA can be stopped [6]. For those IUs who do not qualify for TAS due to persistent low MDA coverage or who must repeat MDA rounds because the critical threshold has been surpassed, a new set of implementation challenges appears. The peer-reviewed literature has not sufficiently addressed these issues. As such, there is a gap in our understanding as to how to guide and assist those IUs when additional MDA rounds must be implemented past the expected 4–6 rounds suggested by the programme [7].

This research aims to respond to that gap in understanding through the development of a tool and process to assist ‘endgame’ IUs in understanding why drug coverage may be persistently low, what specific actions may be undertaken to improve delivery and uptake and how those responsible for delivering MDA may be better supported. Although the issue of low coverage is not a new one, it has become increasingly recognized as the 2020 deadline approaches for LF elimination. Recent reviews on factors associated with coverage and compliance with antihelmintic treatment highlight some of the pertinent issues that need addressing [8–10]. These reviews report similar findings across global and Indian-specific contexts. Notable issues that negatively impact compliance with treatment include fear of side effects, not feeling LF drugs are needed, lack of trust, distributor not coming and taking too many tablets. These reviews focus primarily on findings from the quantitative research portfolio while in this paper we describe the use of a novel survey methodology that combines the use of both qualitative and quantitative data.

Indonesia was chosen as the location for this research. It is the only country in the world with three forms of LF present: *Wuchereria bancrofti*, *Brugia timori* and *Brugia malayi*. Indonesia has participated in the GPELF since 2002, using Albendazole and Diethylcarbamazine in yearly MDA to endemic districts. Across the archipelago, a variety of stages of the LF elimination programme exist–those completing mapping, some just beginning MDA, others moving onto post-TAS surveillance and increasingly, more IUs are applying for TAS.

The process presented in this paper can be described as implementation research (IR). By its design implementation research follows a systematic process that begins with close collaboration between the research team, stakeholders and program implementers to identify a problem related to healthcare delivery and through research finds feasible solutions to improve delivery and access [11]. This paper describes the development of a tool using micronarratives to identify the bottlenecks related to LF drug delivery and drug uptake and the use of that survey to identify feasible recommendations for use in LF endemic communities in two endgame districts in Indonesia. The research also describes how the district health offices used these recommendations in the implementation of an additional MDA round and how that impacted reported drug coverage rates. Finally the implications of this research for LF elimination programmes with IUs in the endgame stages will be discussed.

## Materials and Methods

### Selection of the research sites

Following recommendations from the National LF Programme in Jakarta, two districts were selected as research sites: Depok City and Agam District. Both sites had completed multiple MDA rounds and were entering the endgame stage of their elimination programmes.

Depok City is part of the greater metropolitan areas known as Jabodetabek (Jakarta, Bogor, Depok, Tangerang and Bekasi), which has a population greater than 28 million people, making it one of largest metropolis areas in the world. Depok City is located in West Java province, with a population of 1.75 million in 2010. LF species in this area is *W*. *bancrofti* and the mf rate was recorded as 1.83% (Ministry of Health Indonesia). In 2013, Depok City had completed five rounds of MDA to the whole IU, with coverage rates varying between 46% and 84%, per district calculations. In 2013, mf rates in the spot check and sentinel sites were 0% and the city health department applied to the National LF Programme to implement the TAS. They were denied due to persistent low coverage below 65% in all five previous rounds, using standardized census estimates as the source of total population data, and they were instructed to conduct a further three MDA rounds.

Agam District is located on the western coast of Sumatra, roughly 1200 km from Banda Aceh, site of the Boxing Day tsunami in 2006. In 2010, Agam had a population of just over 450,000 living in both urban and rural areas. LF species in this area is *B*. *malayi* and the mf rate was 8.06% at the beginning of the elimination programme (Ministry of Health Indonesia). Agam District conducted five MDA rounds by 2011 with an average epidemiological coverage rate of 78.2% for the entire IU. The reported drug coverage for these five rounds ranged from 89.6% to 96.7% based on District Health Authority data. Therefore, based on the achieved coverage rates for MDA in Agam and sentinel and spot-check site data assessed as <1% microfilaremia rates, the district qualified for a TAS in 2012. In total, 1315 students from 35 primary schools in all 16 subdistricts were included in the sample. From these, 102 Brugia Rapid tests were positive (from 28 primary schools) (Ministry of Health Indonesia). As a result, Agam District did not qualify to stop MDA and was required to continue MDA for an additional two years (2013, 2014).

### Questionnaire development

The survey tool developed during the course of this research was rooted in the use of a micronarrative or a brief story reflecting personal experiences with the most recent MDA. Unlike Knowledge, Attitudes and Practice (KAP) surveys, the majority of the survey questions related to this specific experience or story. In order to solicit a story, the respondent was asked a specific ‘prompting’ question, like “Tell me what happened after you received the drugs for LF?” Following the respondent’s story, a series of closed questions related to that specific experience were asked, including details about the story participants, the location, the outcome (swallowed the LF pills or not) as well as related emotions.

The micronarrative survey is based on the recognition that participation with MDA is a social process, rather than a strictly individual one. As such, an individual’s direct and indirect experiences with the MDA and with the people associated with MDA will be most revealing about how the implementation of MDA can be improved. One of the important advantages of working with micronarrative is that it does not constrain the respondent to provide information within a tightly prescribed framework of questions and answer options. Storytelling provides a mechanism to explore both expected and unexpected themes, using the respondent’s personal experience as the reference point for subsequent closed questions. Because the use of micronarrative combines the range and depth commonly seen in qualitative research methodologies with the accuracy and precision of cross sectional surveys, it offers a range of analytical possibilities that will be explored in a subsequent publication.

Development of the survey tool was done together with stakeholders and health staff from both districts. Through a series of workshops relevant themes known to be associated with MDA outcomes were identified. The conceptual model used to guide this research used the outcome of taking LF drugs (e.g. compliance) as a function of the interactions between the deliverer, the endemic community member and the MDA setting itself. In actuality, two survey tools were created–one to address the experiences of those involved in the drug delivery and one for the endemic community member receiving the LF drug. This paper presents the survey tool and results for the endemic community survey.

Prior to the baseline survey, the questionnaire was tested in Depok City with 40 community members using enumerators from the Center for Health Research at the Universitas Indonesia in an area outside of the selected research sample. Changes to the questionnaires were made based on this test. After the implementation of the baseline survey and prior to the start of the endline survey, enumerators, the research team and the district health team provided inputs for further refinement of the survey instrument. Some basic changes were made to the overall format, however none of the outcome variables of interest were altered. The final survey tool included the following components: socio-demographic information, a prompt to elicit a specific story related to the last MDA respondents participated in (e.g. “tell me what happened the last time you were offered the LF drugs”), questions related to that experience (side effects, person distributing the drug, reported drug taking behavior), and attitudes towards the MDA, the LF drug, and the perceived drug taking behavior of the household and community.

### Data collection

The EPI cluster survey design was used to calculate the number of clusters in each district (proportionate to population size) for the endemic community surveys (n = 406 in each research site). The sample size was calculated on the following criteria: an anticipated population proportion of 90% with a confidence level of 95% and absolute precision of 5%. The required sample size for these parameters was 138 persons. From four previous similar LF surveys carried out in Indonesia, the intra class correlation coefficient was calculated as 0.235. Using a cluster size of 7, the design effect for this survey was set at 2.41. As a result, the necessary sample size was 333 persons (138 x 2.41). A buffer of 20% was added in the event of refusals and/or incorrectly administered questionnaires. The total sample size required for the survey in each location was 406 persons, or 58 clusters of 7 respondents. Henderson and Sundaresan (1982) recommend a minimum of 30 clusters to ensure that the sample has a normal distribution [12]. The basic sampling unit is the household, rather than the individual. Households were randomly selected at the village level (throwing a pen and walking in the direction of the first house). At the household level, one person was identified through a random selection of all household members present at the time the enumerator visited. One person per household was interviewed. Only those above the age of 15 years were included in the sample.

In both sites, locally based enumerators were selected and trained by Universitas Indonesia researchers on the survey methodology. All questionnaires were administered to respondents by these trained enumerators. This sampling frame and methodology was used for both the baseline and endline surveys.

### Data analysis

For both the baseline and endline surveys, data was double entered using Epi-Info and then transferred for analysis to STATA 14. Data was checked for response bias, and range and consistency checks were completed. Data was adjusted for the cluster effect and was weighted for sex using district population statistics as a reference. Univariate and bivariate analysis informed the construction of multivariable models for outcomes of interest that included: receipt of LF drug, reported drug taking behavior (e.g. compliance) in the last MDA and previous drug taking behavior (e.g. history of having taken LF drugs during any MDA round). In the baseline survey, a multivariable model testing the outcome of “compliance in the story” was done; this model was not constructed in the endline survey. Backward elimination was applied to remove factors from the model that were not significant at the level of 5%. Only the significant predictors for each outcome were retained in the models. However age was selected *a priori* and retained in each model regardless of its significance level. The adjusted Wald test was used for all multivariable models. This paper presents results from the closed questions in the survey. The analysis of the micronarratives will be discussed separately.

Analysis of the survey results for both the baseline and endline surveys was done in close collaboration with the district health authority in both sites. This process facilitated ownership of the data and its results by those responsible for implementation of the MDA at the district level. A wider range of stakeholders was consulted to discuss research findings and resulting recommendations in a series of workshops in both locations.

### Timing of the research

Prior to the start of the research, the last MDA round in Agam District occurred in November 2013 and the baseline survey was conducted there in December 2013. In Depok City, the last MDA round had been conducted in 2013, after which time the District health authority applied for TAS and while waiting for the response ceased MDA activities. The baseline survey was conducted in Depok City in January 2014; roughly one year after the last MDA was conducted. Analysis of survey results was performed in March and April 2014. Presentation and discussion of results at the district health offices was carried out in September 2014, followed by one technical visit to each site by one member of the research team to assist with the incorporation of the research recommendations in the upcoming MDA. Flowcharts were developed for use by drug distributors in both research sites and were finalized during the technical meetings. MDA rounds in both locations were carried out in November 2014 and the endline surveys were performed in both locations within two months of the end of that MDA. Results were discussed at a workshop with the district health authorities in June 2015 and presented to the National LF Elimination programme in Jakarta.

### Ethics statement

The Faculty of Public Health, Universitas Indonesia gave ethical clearance for both the baseline and endline surveys. All questionnaires were anonymous and no personally identifying information was collected. Informed consent from the respondent was obtained prior to the start of the data collection. Eligible respondents were 15 years and above and each respondent gave their own consent in writing to participate in the survey after being informed about the questionnaire, the time required for participation in the survey and understanding whom to consult if there were any additional questions. At the end of the interview, survey respondents were offered a small pencil case for their participation in the research and a leaflet on LF with Universitas Indonesia details, according to Indonesian ethical requirements.

### Definitions used

Definitions related to persons receiving LF drugs and persons taking LF drugs vary significantly in the peer reviewed literature and in the field [8–10]. In the reality of MDA, directly observed treatment is not always implemented on the ground during MDA, and as a result, the WHO definition of drug coverage may not always reflect the true rate of those who took the treatment.

Because of the heterogeneity of the definitions used, this research uses the following definitions. Coverage is defined as the percentage of targeted persons who receive MDA medications and compliance refers to the WHO definition for drug coverage, specifically, the percentage of a targeted population who ingest the medication [8, 13].

## Results

### Characteristics of survey respondents in baseline and endline surveys

A total of 401 questionnaires were accepted for analysis from Agam District and 405 questionnaires from Depok City in the baseline survey (n = 806). In the endline surveys, 405 questionnaires were accepted for analysis from Agam District, and 406 from Depok City (n = 811). Both rounds had similar demographic distributions with the exception of occupation. There were more private workers in the endline survey as compared to the baseline survey (Table 1). Housewives represented the highest proportion of professions recorded across all surveys (35% overall). Age distribution was similar between baseline and endline survey rounds (p = 0.879) with 13% of respondents under the age of 25 years, 24% between 26–35 years, 25% between 36–45 years, 19% between 46–55 years and 19% above the age of 56 years. Education was also similar across the two survey rounds (p = 0.445) with most respondents having completed secondary school (37%). Ten percent had not completed primary school or had never attended school across all surveys. There were some variations in demographics between Depok City and Agam District in terms of education level and occupations, but this was expected due to inherent urban and rural characteristics.

**Table 1:** Demographic characteristics of the sample in Agam District and Depok City.

	Agam District				Depok City			
	Baseline unadjusted	Baseline adjusted* (n = 401)	Endline unadjusted	Endline adjusted* (n = 405)	Baseline unadjusted	Baseline adjusted* (n = 405)	Endline unadjusted	Endline adjusted* (n = 406)
Male	96 (24%)	201 (50%)	103 (25%)	203 (50%)	91 (23%)	204 (50%)	61 (15%)	204 (50%)
Under 25 years	51 (13%)	52 (13%)	61 (15%)	65 (16%)	47 (12%)	53 (13%)	26 (6%)	37 (9%)
26–35 years	99 (25%)	92 (23%)	95 (23%)	85 (21%)	99 (25%)	93 (23%)	120 (30%)	122 (30%)
36–45 years	95 (24%)	104 (26%)	87 (21%)	89 (22%)	112 (28%)	105 (26%)	120 (30%)	106 (26%)
46–55 years	76 (19%)	68 (17%)	72 (18%)	65 (16%)	77 (19%)	77 (19%)	90 (22%)	89 (22%)
56 years and above	80 (20%)	84 (21%)	91 (22%)	101 (25%)	66 (16%)	77 (19%)	50 (12%)	52 (13%)
Private worker	38 (9%)	59 (15%)	52 (13%)	72 (18%)	67 (17%)	101 (25%)	84 (21%)	165 (41%)
Informal worker	124 (31%)	164 (41%)	85 (21%)	116 (29%)	55 (14%)	70 (17%)	6 (1%)	21 (5%)
Housewife	188 (47%)	123 (31%)	212 (52%)	143 (35%)	225 (56%)	145 (36%)	277 (68%)	161 (40%)
Have not completed primary school	62 (15%)	60 (15%)	66 (16%)	72 (18%)	29 (7%)	20 (5%)	27 (7%)	16 (4%)
Completed secondary school	203 (51%)	138 (35%)	221 (55%)	104 (26%)	251 (62%)	180 (45%)	276 (68%)	175 (43%)
Completed a diploma / university degree	36 (9%)	34 (9%)	24 (6%)	61 (7%)	78 (19%)	81 (20%)	64 (16%)	81 (20%)

*Adjusted for sex and clustering effect

Both survey rounds had proportionately (relative to the population) more females in the sample, likely due to the interview scheduled during the daylight hours in consideration of security and logistical constraints. As a result, the sample was adjusted for gender for analysis purposes. In addition the data was also adjusted for the effect of the cluster design. All data presented here use the adjusted results.

### Baseline survey results

Respondents were asked in their narrative prompt to respond to the following question, “Earlier you mentioned that you had received the LF drug during MDA. Could you tell me about it, what happened?” Most of the recorded stories were related to receiving and taking the LF drugs (53%), receiving the drugs (28%) or taking the drugs (16%). A sample micronarrative from a woman in her thirties in Agam District:

“In the morning, there was a general announcement from the mosque next door to my house that there would be a drug distribution for filaria at the integrated health post (Posyandu). When I got there, the midwife asked me how old I was, and then she gave me the drug and told me to take it before going to sleep. So I went home, and at night that day, I took the drugs.”

Half of the survey respondents reported that they had received LF drugs from a community health worker (50%) whilst over a quarter received LF drugs from a family member, friend or neighbor (27%). Sixty-three percent reported that they took all of the pills they were given while 8% reported that they took only some of the pills. Most respondents indicated “myself” as the greatest influence on their decision to take the pills (77%), followed by the health worker and community health worker (10%). Nearly half (49%) reported no side effects after taking the treatment.

Women were less likely than men (AOR = 0.53) to have complied with treatment in the last MDA (p = 0.011). Predominant reasons for noncompliance in the last MDA included being pregnant (4% of total noncompliers), too old (4%), sick at the time of distribution (17%), taking other drugs (12%) and lack of information (19%). In the Indonesian eligibility guidelines for MDA at the time of the baseline survey, breastfeeding women and people above the age of 65 years were excluded from treatment.

Specific questions related to the last MDA included: where the LF drugs were received, awareness about MDA, knowledge of other family members’ compliance with MDA and one question related to knowledge of the cause of LF. In Agam District, 71% of respondents were aware of the MDA before it occurred, compared to 67% in Depok City. Most people in Agam District received the LF drugs inside their homes (79%) confirming the house-to-house distribution method preferred in this area. In Depok City, 56% of respondents received their LF drugs inside their house reflecting the higher use of distribution posts here due to the high population density, presence of apartment buildings and the mobile nature of an urban population. Respondents were asked if they knew of anyone else in their household who had complied with the LF drugs: in Agam District 75% knew someone in their household, compared with 69% in Depok City. In both locations, around a quarter of respondents identified worms (22% in Agam District; 25% in Depok City), and mosquitoes (31% in Agam District; 48% in Depok City) as the cause of LF.

Respondents were asked some attitudinal questions about MDA and LF drugs. In Agam District, more respondents cited that LF drugs were safe (73%), compared to Depok City (62%). However in both research locations, a majority of respondents believed that MDA was very important for their health (85% in Agam District and 77% in Depok City).

Multivariable logistic regression models were created for four key outcomes of interest (Table 2): ever complied with LF drugs, ever received LF drugs, reported compliance in the last MDA offered and reported compliance described in the story.

**Table 2:** Multivariable models for Baseline survey (2014)^.

		Unadjusted values		Adjusted values	
	N	OR (95% CI)	P-value	AOR (95% CI)	P value
**Ever received LF drugs during MDA (N = 804)**					
*Sex*					
Male	405	1.00		1.00	
Female	399	2.56 (1.7–3.9)	<0.001	3.02 (1.7–5.5)	0.001
Age**					
15–25 years	103	1.00		1.00	
26–35	185	1.92 (1.0–3.7)	0.050	2.20 (1.0–4.7)	0.041
36–45	209	3.12 (1.6–5.9)	0.001	3.94 (1.8–8.7)	0.001
46–55	146	5.67 (2.3–14.3)	<0.001	7.38 (2.5–21.9)	0.001
56+	159	2.12 (1.2–3.8)	0.013	2.82 (1.2–6.7)	0.019
**Ever taken LF drugs (N = 631)**					
*Location*					
Agam	323	1.00		1.00	
Depok	308	0.40 (0.3–0.7)	<0.001	0.36 (0.2–0.7)	0.003
*Perceived safety of the LF drug*	631*				
Yes, drugs are safe		1.00		1.00	
No, not safe		0.55 (0.4–0.7)	<0.001	0.60 (0.5–0.8)	<0.001
Knows another household member who took LF drugs**					
Yes	456	1.00		1.00	
No	148	0.17 (0.1–0.3)	<0.001	0.18 (0.1–0.3)	<0.001
Don’t remember	26	0.37 (0.2–0.9)	0.036	0.30 (0.1–0.9)	0.024
Location where drugs were received**					
Inside the house	429	1.00		1.00	
Outside the house	164	1.68 (1.0–2.8)	0.047	2.74 (1.4–5.4)	0.004
*Media stories were informative and helpful*					
No	289	1.00		1.00	
Yes	342	2.02 (1.4–2.9)	<0.001	2.10 (1.3–3.3)	0.002
**Compliance in the last MDA (N = 631)**					
*Sex*					
Male	287	1.00		1.00	
Female	344	0.90 (0.6–1.3)	0.579	0.53 (0.3–0.9)	0.011
Perceived importance of LF drugs for health**	629*				
Yes		1.00		1.00	
No		0.55 (0.4–0.7)	<0.001	0.62 (0.5–0.8)	0.002
Perceived safety of drugs**	625*				
The drugs are safe		1.00		1.00	
No, the drugs are not safe		0.53 (0.4–0.7)	<0.001	0.60 (0.5–0.8)	<0.001
*Health status in the last MDA e*.*g*. taking other medications at the time of MDA**					
Not taking other drugs	570	1.00		1.00	
Yes, taking other drugs	58	0.13 (0.1–0.3)	<0.001	0.12 (0.1–0.3)	<0.001
Never taken LF drugs before**					
Has taken LF drugs before	375	1.00		1.00	
Never taken LF drugs before	238	0.29 (0.2–0.4)	<0.001	0.37 (0.2–0.6)	<0.001
**Reported compliance in the story (N = 631)**					
Location**					
Agam	322	1.00		1.00	
Depok	308	0.45 (0.3–0.7)	0.001	0.44 (0.2–0.8)	0.009
Sex**					
Male	286	1.00		1.00	
Female	344	0.86 (0.6–1.3)	0.435	0.48 (0.3–0.9)	0.015
*Aware of MDA prior to drug distribution*					
No	60	1.00		1.00	
Yes	571	1.61 (0.9–2.8)	0.097	2.59 (1.0–6.7)	0.048
Perceived common good as reason for compliance**					
No	326	1.00		1.00	
Yes	304	2.69 (1.8–3.9)	<0.001	1.50 (1.1–2.1)	0.019
*Perception of taking LF drugs to be healthy*					
No	122	1.00		1.00	
Yes	509	14.05 (6.9–28.8)	<0.001	10.74 (5.1–22.6)	<0.001
*Fear of side effects*					
No	539	1.00		1.00	
Yes	92	0.28 (0.2–0.4)	<0.001	0.24 (0.11–0.5)	<0.001
*Because other people took the drugs*					
No	542	1.00		1.00	
Yes	89	3.08 (1.5–6.4)	0.003	2.27 (1.1–4.7)	0.030
*Influence of the drug packaging*					
No	556	1.00		1.00	
Yes	75	2.56 (1.3–5.2)	0.010	1.88 (1.0–3.5)	0.048
Influence of the level of information received**	629*				
No		1.00		1.00	
Yes		1.24 (1.1–1.4)	0.004	1.28 (1.1–1.6)	0.014

^Variables with p<0.05 were included in the final models.

*Denotes continuous variable

**Missing values.

Data from the baseline surveys showed that 19% of respondents in Agam District and 24% of respondents in Depok City had never received the LF drugs during any MDA. In the multivariable model (after adjusting for district, education, income and occupation) age and sex were determined to have had an effect on whether or not respondents had ever received the LF drugs. Overall, women were three times more likely to receive the LF drugs as compared to men (AOR = 3.02; p = 0.001). This may reflect the distribution strategies used in both sites where MDA was conducted primarily during the day. Respondents aged 15–25 years were the least likely to receive the LF drugs as compared to respondents aged above 26 years. Those aged between 46–55 years were 7 times more likely to have ever received the LF drugs as compared to respondents aged 14–25 years (AOR = 7.38; p = 0.001).

In the questionnaire, respondents were asked if they had ever taken the LF drug during any MDA offered in the past. Nearly 62% of those who had ever received the drugs had a history of compliance in both research sites, meaning that 38% of those who had been offered the LF drugs had never taken them. These individuals, called systematic noncompliers, can be defined as people who persistently refuse or do not ingest the antifilarial medications over the course of an MDA program [8]. Systematic noncompliers may harbor LF infection and have the potential to contribute to LF resurgence [14, 15]. Factors related to systematic noncompliance in our study included the perception that the LF drugs were unsafe (AOR = 0.6; p<0.001) and not knowing anyone in the household who had taken the LF drugs (AOR = 0.18; p<0.001). Positive associations with a history of having taken the LF drugs included being given the LF drugs outside of the house (AOR = 2.74; p = 0.004) and perceiving media stories to be informative and helpful (AOR = 2.10; p = 0.002).

For compliance in the last MDA, the multivariable model was stratified by location to elucidate if there were differences between the urban (Depok City) and rural (Agam District) datasets. As discussed earlier, the survey was conducted within one month of the last MDA in Agam District, and more than one year after the last MDA in Depok, so we anticipated some differences due to recall of events. The following variables were associated in these analyses. In Depok City, (1) age was not a factor associated with compliance; (2) Working in the private sector had a lower odds for compliance than those who were unemployed (p = 0.006); (3) The perceived importance of LF drugs for health positively influenced drug taking behavior (p = 0.02); (4) Past history of compliance was seen as an important influence, e.g. if respondents had never taken the LF drug, then they were less likely to comply in the last MDA (p = 0.002). In Agam District specifically: (1) Those who were 26–35 years were less likely to comply in the last MDA than the 15–25 year old group (p = 0.013); (2) Working in the private sector had a higher odds for compliance than the unemployed (p = 0.02); (3) Perceived good drug safety positively influenced the decision to comply in the last MDA (p = 0.004).

In the multivariable model for reported compliance in questions related to the story (adjusted for district, age, income, education and occupation) several factors were associated with taking the LF drug. In the stories, the value of “being healthy” had a strong positive influence on compliance. Those reporting that being healthy was an important influence on their decision to take LF drugs were nearly 11 times more likely to report compliance in their stories than those who did not cite “being healthy” as an influence (AOR 10.74; p<0.001). Perceived common good (AOR 1.5; p = 0.019) had a positive influence on compliance, suggesting respondents understood the norm that taking LF drugs benefits the community. This social norm of compliance was also seen in the positive influence of knowing others had taken the treatment in the stories. Those who reported that others taking the LF drugs influenced their own behavior were 2.27 times more likely to report their own compliance in their stories (AOR 2.27; p = 0.030).

Women were less likely than men to have taken the LF drugs in the last MDA (AOR = 0.53; p = 0.011) as well as in the MDA experiences they described (AOR = 0.48; p = 0.015). Pregnancy and breastfeeding (considered as contraindicated in some Indonesian district MDAs) may explain why women were less likely to comply.

### Recommendations for implementation of next MDA round in 2014

After the results were compiled, a series of workshops were held to discuss the results with the District Health teams and to present the findings to relevant stakeholders in both districts and at the national level. Feasible actions to address issues related to coverage and compliance were identified with program personnel from each location. In addition to the workshops, prior to the start of MDA awareness activities in each site, one member of the research team gave a brief technical visit to further discuss the survey results with stakeholders and other district health staff.

In order to improve distribution of LF drugs during MDA in both areas, the primary groups that needed more targeted attention were men and youths between the ages of 15–24 years. At the time of the MDA, women were successfully receiving the drugs in both areas with the distribution strategies in place. In order to widen the reach and to increase men’s participation, it was recommended to consider an approach to MDA that would occur simultaneously at schools, government and private offices, households as well as factories. In order to reach younger persons, use of social media and text messaging were suggested.

For those who had never complied with taking the LF drugs in the past (considered as systematic noncompliers), the findings suggest that they were also unlikely to comply in future MDA rounds, thus continuing their pattern of behavior. As such, it was recommended to develop a method to identify these persons at the start of the drug delivery encounter so that the drug distributors could target them with specific messaging. A flow chart of questions was created for the drug distributors to use. It began with the question, “When was the last time you took the LF drugs during MDA?” Subsequently, the distributor was prompted through a series of questions and responses to aid them in persuading this person to accept LF drugs. As media stories considered as positive and helpful were seen to be associated with compliance, it was recommended to have an intentional media campaign, if possible emphasizing the social norm of compliance, e.g. “I took it with the other people in my family, neighborhood, city.”

Another issue that was identified through the baseline survey was the ineligibility criteria used by drug distributors during MDA. Individuals taking other medications at the time of MDA (namely for hypertension and diabetes), those over the age of 65 years and breastfeeding women were frequently excluded from treatment. It was recommended to the national LF program to increase the upper age limit for MDA eligibility and reinforce the international guidelines regarding exclusion. In addition, messages about drug safety should be used to help promote trust and reduce fear of side effects for communities.

Baseline data revealed that perceived drug safety, number of pills and packaging of LF drugs had an important influence on the decision to swallow the pills. As a result, it was suggested to package the pills with specific messages addressing the following: drug safety (“# million people in Indonesia safely took LF drugs last year”); drug-taking procedure (“take all the drugs at once, preferably with a meal”); ineligibility information (children under 2 years, pregnant women and severely ill persons); benefits of compliance for yourself, your family and community and finally where to go if you need assistance.

Finally in terms of messaging surrounding the next round of MDA, it was recommended that the district health authorities focus their messages on the social norm of compliance (e.g. “everybody is doing it”) and on the safety of LF drugs globally and in Indonesia. It was suggested that messaging regarding side effects continue to be used, with a focus on promoting the message that side effects indicate that the medicine is working. Ancillary benefits to treatment regarding the elimination of intestinal helminthes should also be promoted, particularly in Agam District. Finally MDA should be promoted as a preventive activity, rather than a treatment (“taking it will keep you healthy”). This would counteract the argument some community members made that they were not sick, so therefore did not need to take LF drugs.

### Actions taken by the district health authorities in the 2014 MDA

Because the remit of this project at the outset was primarily to design and test an effective research tool, there was no budget available to assist the districts with their MDA operations. Furthermore, the research team did not monitor the planning or execution of MDA in either site. Both research sites followed through on many of the discussed recommendations, as described here.

In Agam District, the district health office was able to secure additional funding from the local government to implement the two MDA rounds they were requested to complete. Note that with decentralized health financing in Indonesia, many districts are required to fund the operational costs for LF elimination themselves. Based on the recommendations of this study, the district health office in Agam retrained the 4000 community health workers responsible for drug distribution. Promotional media was used, including stickers on government vehicles, additional production of leaflets as well as banners. Prior to 2014, schools had never been approached to aid in the promotion of MDA. After interpreting the baseline survey results with the district health team, teachers were provided with the flowcharts produced by the project. These flowcharts aided teachers in promoting the drug distribution by guiding them to ask their students if they had taken their LF drugs after the recent MDA. In an attempt to better reach men, the district team worked with local factories, distributing the drugs during working hours after securing consent from the factory management.

Depok City was unable to secure additional local financing for its repeated MDA rounds. As a result the head of the program used every opportunity to integrate LF promotional and educational messages into existing activities. In most district health meetings, the LF program promotes the MDA to those stakeholders present. Using existing primary health care center funds, community health workers participated in “refreshing” activities prior to MDA, where previous training was reviewed. New stakeholder groups were approached, specifically police and army barracks located in the city, private and public hospitals, the Indonesian Association of Midwives, the Indonesian Doctors Association as well as local NGOs to promote and facilitate MDA. In terms of additional promotional activities, a running text billboard ran messages one month prior to the start of MDA and a radio show integrated LF messages into their regular programming. A number to call or text questions was posted and promoted so that the community members could present concerns to the health team. Finally, a small leaflet was produced for inclusion with the drug packages. This provided point of contact information for the drug recipients on how to take the pills. Health staff and community health workers were provided with a Frequently Asked Question (FAQ) sheet to aid with drug distribution.

### Endline survey results

The three primary outcome variables related to compliance showed a marked improvement in the endline surveys relative to the baseline surveys (Table 3). Of those who had ever received LF drugs, Agam in particular showed a marked increase from 81% to 100% of those surveyed. This indicates that Agam District was able to reach significant numbers of new individuals who had never received LF drugs in previous MDA rounds. Specifically this represented an increase in drug receipt across all age ranges, with the highest being a 40% increase for those under the age of 25 years, one of the key target groups identified in the baseline survey.

**Table 3:** Comparison of baseline and endline results.

	Agam District		Depok City		Total	
	Baseline	Endline	Baseline	Endline	Baseline	Endline
*N = total sample*						
Have ever received the LF drug during MDA	80.6%	100%*	76.3%	76.9%	78.4%	88%*
*N = individuals who had ever received LF drugs during MDA*						
Have ever taken the LF drug during MDA	86.1%	92.1%*	72.3%	88.1%*	79.3%	90.4%*
Received LF drug during last MDA	68.7%	90.8%*	69.7%	73.8%	69.2%	82%*
Took the LF drug in the last MDA	66.6%	84.1%*	48.2%	67.3%*	57.3%	77%*
Aware of MDA before receiving drugs	71%	85.8%*	67%	85.6%*	69.1%	85.7%*
Knew someone in the house that had taken LF drugs	75%	84.6%*	69.4%	81.9%*	72.3%	83.5%*
LF drugs perceived as safe	73%	71.9%	62%	66.9%	67.62%	69.79%
Taking LF drugs perceived to be important for health	85%	86.4%	76.8%	88.9%*	81.0%	87.5%*
Reported to have swallowed all of the LF drugs at once	71.6%	82.1%*	53%	66.7%*	62.5%	75.4%*
Not having enough information as the reason cited for noncompliance	18.5%	14.8%	19.9%	8.6%	19.4%	10.9%

*Change in percentage where p<0.05

For those who reported a history of taking LF drugs in the past, the most prominent change was seen in Depok, from 72% in the baseline to 88% in the endline survey. This represents an increase in the uptake by first time compliers. For the total sample, the change in compliers went from 79% in the baseline to 90% in the endline survey, of those who had ever received LF drugs. Table 3 provides the summary of results of key outcome variables for both baseline and endline surveys.

Respondents were asked about their participation in the last round of MDA. Agam District increased the proportion of those receiving LF drugs in the last round by 32% while Depok City’s improvement was 6%. The change in those reported to have taken the drugs in the last MDA was more marked in Depok City from 48.2% in the baseline round to 67.3% in the endline round, representing a 40% improvement between the two survey rounds. In Agam the change was from 66.6% to 84.1%, representing an improvement of 27% for compliance in the last MDA.

Other changes between baseline and endline datasets included key indicators related to the recommendations that were given prior to the 2014 MDA. Awareness of MDA prior to drug distribution increased in both sites, as did the awareness of someone taking the treatment in the household. The message to take all of the pills at the same time appears to have been well communicated in both research locations with a marked improvement from 63% to 75% (p<0.001) overall.

In the multivariable analysis (Table 4), some factors remained strongly associated with compliance as seen in the first survey round. In the multivariable model for compliance in the last MDA, perceived importance of LF drugs for health continued to be strongly associated with compliance (AOR 42.76; p = 0.001). Similarly those who believed that LF drugs were safe were 3.7 times (AOR) more likely to comply in the last MDA round than those who perceived the drugs as dangerous (p = 0.027). As in the baseline models, those who did not know anyone else in their household who took the drugs were less likely (AOR 0.16) to comply in the last MDA round than those who did (p<0.001).

**Table 4:** Multivariable models for Endline survey (2015)^.

		Unadjusted values		Adjusted values	
	N	OR (95% CI)	P value	AOR (95% CI)	P value
**Ever received LF drugs during MDA (N = 811)**					
*Age*					
15–25 years	99	1.00		1.00	
26–35	208	1.96 (0.8–4.65)	0.128	2.12 (0.8–5.3)	0.107
36–45	195	3.21 (0.9–10.6)	0.056	3.22 (1.1–9.1)	0.028
46–55	155	4.09 (0.9–16.8)	0.05	4.47 (1.2–16.2)	0.023
56+	154	2.02 (0.6–6.5)	0.237	1.68 (0.6–4.4)	0.291
*Occupation*					
Housewife	304	1.00		1.00	
Private	237	0.29 (0.1–0.6)	0.002	0.28 (0.1–0.6)	0.002
Informal	137	3.51 (0.6–20.9)	0.166	3.74 (0.7–20.1)	0.123
Other	133	0.35 (0.1–0.9)	0.029	0.57 (0.2–1.3)	0.196
**Ever taken LF drug (N = 717)**					
*Location*					
Agam	405	1.00		1.00	
Depok	312	0.64 (0.3–1.2)	0.148	0.44 (0.2–1.0)	0.056
*Age*					
15–25 years	77	1.00		1.00	
26–35	181	0.30 (0.1–0.9)	0.038	0.19 (0.0–1.2)	0.082
36–45	179	0.37 (0.1–1.1)	0.061	0.07 (0.0–0.4)	0.006
46–55	145	0.77 (0.2–2.5)	0.652	0.16 (0.0–1.2)	0.078
56+	135	0.4 (0.1–1.4)	0.157	0.15 (0.0–1.2)	0.075
Income**					
Less than minimum wage	350	1.00		1.00	
Equal to or more than minimum wage	338	0.68 (0.3–1.5)	0.330	0.23 (0.1–0.7)	0.007
*Perceived safety of drugs*					
Dangerous	45	1.00		1.00	
Neutral	172	5.00 (1.8–13.9)	0.002	3.66 (1.0–13.4)	0.050
Safe	500	21.56 (8.3–56.4)	0.000	6.33 (1.7–22.9)	0.005
*Aware of MDA prior to drug distribution*					
Yes	614	1.00		1.00	
No	103	0.29 (0.1–0.7)	0.003	0.30 (0.1–0.8)	0.020
*Perceived importance of LF drugs for health*					
Unimportant for health	21	1.00		1.00	
Neutral	69	13.77 (3.1–60.8)	0.001	23.04 (3.7–142.6)	0.001
Important for health	627	67.01 (14.7–305.6)	0.000	42.63 (7.2–252.6)	<0.001
Influencing the decision to comply**					
Myself (no outside influence)	423	1.00		1.00	
Others	288	3.34 (1.6–7.2)	0.002	2.65 (1.1–6.5)	0.034
*Knows the deliverer*					
Yes	640	1.00		1.00	
No	77	0.22 (0.1–0.5)	0.001	0.18 (0.1–0.5)	0.001
*Knows another household member who took LF drugs*					
Yes	598	1.00		1.00	
No / Don’t know	119	0.07 (0.03–0.2)	<0.001	0.11 (0.0–0.3)	<0.001
Possible negative or positive effects of MDA on health**					
Less important	219	1.00		1.00	
Important	265	1.70 (0.5–5.4)	0.362	1.37 (0.3–5.5)	0.656
Very important	214	0.21 (0.1–0.5)	0.001	0.27 (0.1–0.8)	0.019
**Compliance in the last MDA (N = 665)**					
*Location*					
Agam	365	1.00		1.00	
Depok	300	0.39 (0.2–0.7)	0.001	0.26 (0.1–0.5)	<0.001
*Age*					
15–25 years	70	1.00		1.00	
26–35	166	0.51 (0.3–1.0)	0.060	0.60 (0.2–2.0)	0.401
36–45	174	1.05 (0.5–2.1)	0.885	1.26 (0.4–4.2)	0.711
46–55	134	1.93 (0.9–4.2)	0.096	3.76 (1.0–13.6)	0.044
56+	121	1.42 (0.6–3.5)	0.440	3.06 (0.7–13.4)	0.135
*Perceived importance of LF drugs for health*					
Unimportant for health	21	1.00		1.00	
Neutral	57	11.03 (1.2–98.6)	0.032	30.83 (3.6–263.9)	0.002
Important for health	587	34.01 (3.9–296.8)	0.002	42.76 (4.8–380.4)	0.001
Perceived safety of drugs**					
Dangerous	42	1.00		1.00	
Neutral	156	2.59 (1.0–6.6)	0.045	1.74 (0.5–5.9)	0.370
Safe	466	8.07 (3.1–20.8)	<0.001	3.73 (1.2–11.9)	0.027
Cause of LF**					
Worms	126	1.00		1.00	
Mosquitoes	230	0.81 (0.4–1.5)	0.494	0.56 (0.2–1.3)	0.187
Don’t know	257	0.37 (0.2–0.8)	0.016	0.25 (0.1–0.6)	0.005
Other	29	0.75 (0.3–2.3)	0.615	0.42 (0.1–1.6)	0.204
Possible negative or positive effects of MDA on health**					
Less important	199	1.00		1.00	
Important	251	1.31 (0.6–3.1)	0.529	1.02 (0.5–2.2)	0.960
Very important	200	0.29 (0.1–0.6)	0.001	0.42 (0.2–0.9)	0.016
*Length of stay in the survey district*					
<2 years	30	1.00		1.00	
2+ years	635	0.66 (0.2–2.2)	0.499	0.06 (0.01–0.3)	0.001
*Knows another household member who took LF drugs*					
Yes	565	1.00		1.00	
No	100	0.10 (0.1–0.2)	<0.001	0.16 (0.1–0.3)	<0.001

^Variables with p<0.05 were included in the final models.

**Missing values.

In addition, some new factors emerged that were associated with compliance. In the model for compliance in the last MDA, length of stay in the region less than two years was associated with higher odds of compliance than those who had lived in the area for more than two years (p = 0.001). Additionally those who did not know a cause of LF were less likely to have complied in the last round of MDA (AOR 0.25; p = 0.005) than those who knew that worms caused the disease in the body.

In the model for “having ever taken LF drugs” some new factors emerged as well. In terms of the identity of the drug distributor, respondents who knew the drug deliverer were more likely to comply compared with those who did not know the deliverer (p = 0.001). External influence on the decision to comply remained strong. Respondents who cited an external influence on their decision to comply were 2.6 times (AOR) more likely to have ever taken LF drugs compared to those who reported no outside influence (p = 0.034).

Table 5 summarizes the key factors that were positively associated with complying with the LF treatment from both the baseline and endline surveys.

**Table 5:** Summary of factors positively associated with taking the LF treatment.

Ages 36–45 years^; 46–55 years^
Male*
Earns less than the monthly minimum salary^
Lives in the area less than 2 years^
Not taking other medication at time of MDA*
Takes LF drugs before*
Knows the deliverer^
Receives the drugs outside of the house*
Knows another household member who took LF drugs*^
Aware of MDA prior to distribution*^
Media stories are informative and helpful*
Perceives LF drugs to be safe*^
Perceives importance of LF drugs for health*^
Perceives the common good as a reason to take the LF drugs*
Not afraid of side effects*
Because other people took the LF drugs*
Influences others to take the LF drugs^

*Baseline survey

^Endline survey

## Discussion

This research demonstrates that both drug distribution and ingestion of pills by the community can be improved when the issues affecting MDA delivery are identified using appropriate tools and processes and then are acted upon by the district health teams. The delivery of MDA was assessed in two research sites in Indonesia where there had been a history of insufficient coverage rates and persistent LF transmission. Post-MDA baseline surveys were conducted in endemic communities in late 2013, early 2014 in both districts. Based on the results of those surveys, the district health teams worked together with the research team to identify feasible recommendations to apply to their 2014 MDA programs. The research team did not provide any supplemental funds for MDA operations and both districts were responsible for implementing the identified recommendations. Results from the endline survey conducted shortly after the 2014 MDA showed that there was a marked improvement in coverage and compliance as well as in other key indicators. The baseline and endline surveys described in this research used a novel approach–asking people to recount their experiences with MDA in micronarratives. These stories provided the foundation to assess MDA programs through the perspectives of the people expected to ingest the LF drugs. The effectiveness of the micronarrative survey combined with the close collaboration with the district health teams has been shown in the overall improvement of drug delivery and compliance.

As the 2020 elimination goal approaches, increasingly districts will reach the transmission assessment process and post-MDA surveillance period. The decision to begin these processes will be made based on the reported drug coverage data submitted by the implementation units (IUs). For programs where directly observed treatment (DOT) is not routinely used, distributed drugs may not necessarily be consumed. As a result, reported drug coverage rates will reflect distributed drugs as opposed to ingested drugs. Because of this coverage-compliance gap [9] in reporting, it is possible that transmission assessment surveys (TAS) may begin prematurely in some areas [16].

For implementation units where reported drug coverage rates are sufficient over the course of the MDA rounds, the IU will qualify for TAS. The district health personnel approach the first steps of TAS hopeful of an outcome that will allow MDA to stop. However if TAS reveals ongoing LF transmission, then the district must agree to continue administering additional MDA rounds as per the international guidelines [13]. Reasons for ongoing LF transmission are varied, including evidence that significant levels of noncompliance with taking LF drugs can maintain a reservoir of LF infection [14, 15]. This level of noncompliance may be due to individual behavior, drug distribution issues or misclassification in reporting. It is important to note that health staff may not be aware of the coverage-compliance gap in their own reporting and so a “negative” result in the first round of the TAS can come as a surprise. In both Agam District and Depok City, district health staff reported feeling discouraged and confused by pre-TAS and TAS results that indicated additional MDA rounds were required.

Because of decentralized planning and funding in Indonesia, the district health staff in both study areas were required to request more resources from district government budgets in order to carry out these unscheduled additional MDA rounds. With this, success in future MDA rounds became of paramount importance. In order to ensure improved coverage in subsequent MDA rounds, the district LF programs needed input and guidance to understand the real impact of their past MDA activities–what groups were missed; where should attention be focused; what had worked; what needed improvement? This research provides a systematic process and innovative data collection tool to district or national programs to help them gain perspective on their operational MDA from the perspective of both the community and the drug distributor.

One of the primary aims of this research was to test the acceptability and feasibility of a novel methodology–the micronarrative survey–as applied to the MDA environment. Unlike routine coverage surveys, the tool developed in this research provided a deeper understanding of the MDA, beyond questions of drug receipt, drug compliance and knowledge about LF, to eliciting people’s direct experiences with the program. In this context, the micronarrative survey proved to be a powerful tool to reveal themes that were associated with influencing compliance. Some of the factors identified through this research are new and further our understanding of why people swallow the LF drugs; while other findings echo factors from research in other global studies.

Some of the themes associated with coverage and compliance detected in this research are consistent with results from other studies, contributing to the potential of this methodology to identify why people receive and take LF drugs (and why they do not). For example, advanced knowledge of the MDA before drugs were distributed was an important factor associated with compliance in this research, echoing results from many programs in both the Asian and African regions [8, 17]. Perceived poor safety of the LF drugs was shown to have a negative effect on compliance in both the baseline and endline surveys, revealing a lack of trust in the program. The lack of trust or misconception about the MDA program has been cited as an attitude associated with low compliance in a recent systematic review in India [9]. In the endline survey, respondents who did not know their drug distributor or trust them were less likely to have received LF drugs in the last MDA. Similarly results from Sri Lanka have shown that compliance is positively affected when the community knows their drug distributor [18] and conversely in India low acceptability of the drug distributor was determined to have a negative effect on compliance [19].

This research identified two groups who were less likely to comply with LF treatment: females and the youngest age group (15–25 years). A study from Egypt also identified that being male as well as having older age were both positively associated with compliance [20]; while research in Haiti demonstrated systematically low compliance rates in women [21]. In Indonesia, the national LF programme originally recommended that breastfeeding women should be considered as ineligible for MDA; however this position has recently changed. These changes to eligibility criteria can take time to filter down to the drug distributor where they are operationalized.

Throughout each step of the process of implementation research, from identifying the important issues related to MDA, to designing the survey tool and reviewing the results, the research team worked closely with district health staff from both Agam District and Depok City. This process fostered ownership of the data and its results, both positive and negative. Through the various workshops, both district health teams had multiple opportunities to share their own experiences and challenges as well as their own best practices with each other. This exchange empowered both teams to enact change rather than react defensively when faced with the research results.

By reviewing the baseline survey results together, the district health staff was able to discern which operational recommendations would be feasible within their own contexts and existing district health budgets. Some of the actions based on the baseline results that were used in the districts included: (1) an approach to MDA that occurs simultaneously in several locations (schools, government offices, factories); (2) the use of social media to reach younger people; (3) engaging teachers and schools to promote MDA and compliance with MDA; (4) reinforcing messages that LF drugs are safe and that the combination of drugs received during MDA should be taken all at once; (5) encouraging directly observed treatment; (6) utilizing drug packaging as an opportunity to inform people; (7) promoting messages that encourage the social norm of compliance e.g. “everyone is taking it”; (8) promoting messages that reinforce the positive healthy impacts of LF treatment, lessening the primary emphasis on side effects; (9) ensuring that the community is aware of MDA before it begins; (10) aiding the drug distributors to address key questions arising from the community and to identify never compliers at the start of the drug distribution encounter.

Both research areas adapted these recommendations to their local contexts and were able to significantly improve the coverage and compliance in their added MDA rounds. For Agam, the most significant change was the shift from 80% at baseline to 100% coverage (e.g. drug receipt) by surveyed community members in the endline survey. This change represents new people who were reached in the 2014 MDA, most likely the result of improved drug distribution efforts using schools and factories for the first time. For Depok, overall distribution to eligible persons between the two rounds remained mostly unchanged; however district efforts resulted in an increase of first time compliers who took LF drugs for the first time during the 2014 MDA. This was likely accomplished through the wide scale integrated approach that the program used to reach new community members, stakeholders and key groups.

These study results are promising in a few key areas. They show that LF program personnel at the district level when provided with relevant information have the influence and willingness to alter their MDA so as to improve both coverage and compliance. It cannot be assumed that district programs always have the support and information that they need to adequately address deficiencies in their MDA delivery and in some contexts, a more targeted approach may be required. Although this specialized technical support at the district (IU) level seems expensive and unsustainable given the scope of the global LF elimination program, it has the potential to make a significant impact. In twelve months, the two LF programs involved in this research were able to make substantial changes to their MDA delivery without additional funding using a deeper understanding of their MDA to guide the necessary changes. Although beyond the remit of this research, understanding the long term cost savings of implementing a more tailored approach to MDA as described here may have important implications for the global LF elimination programme in terms of costs averted from failed TAS and additional MDA rounds.

Finally, the novel micronarrative research tool, which focused on collecting people’s experiences with the MDA program, was shown to be capable of producing valuable insights into factors associated with coverage and compliance that were then translated into action with a measurable impact on program goals. Much of the social research related to LF elimination in the past has focused on questions of knowledge, attitudes and practice (KAP). Compliance with LF is a social activity, whether taken publicly at a distribution post or at the dinner table. The importance of these social influences is often not captured in routine KAP and coverage studies. As a result, promotion of MDA has tended to focus on increasing knowledge at the individual level as opposed to generating a social movement in the community to eliminate a disease and to promote health. This tool has demonstrated the value and potential of taking social research about health behavior (in this case receiving and ingesting LF drugs) outside of the narrow realm of KAP into one where the aim is to understand what has happened to people and how those experiences shape the decisions they make. That said, the scope of this tool and process could be applied to other areas, outside of LF elimination, to explore people’s experiences with other questions of drug adherence, the evaluation of health service provision, or monitoring and evaluation of new initiatives or tools.

### Limitations

There are some the limitations in our research that are worth noting. The EPI methodology when applied to the context and combined with the logistical constraints in Agam District and Depok City resulted in an overrepresentation of females in the survey total. Research enumerators limited their household visits to daylight and early evening hours, thereby missing some males who work outside the home. To control for this, we weighted our sample according to the demographics in those two areas. Another limitation related to the data collection related to the start of the rainy season in January. This resulted in some delays in our data collection which meant that the results were not available to the district health teams before March / April, after the time when the district budgets were allocated. Based on the results of our endline survey, it appears that this delay did not affect the uptake of recommendations that showed an impact on coverage and compliance.

### Conclusions and recommendations for future action

District health staff may not be aware of the actual compliance (drug ingestion) in their area due to use of different definitions in reporting or metrics used in calculation [10] therefore they may be surprised when they do not fulfill pre TAS and TAS requirements. During the 4–6 year course of MDA, it is therefore recommended to carry out at least one coverage survey to assess the presence of a coverage-compliance gap in IUs especially where directly observed treatment is not enacted. Furthermore in those areas where coverage has known to be problematic or a considerable coverage-compliance gap is known, district health staff may not always have the tools or information to understand how to improve their MDA. Therefore it is recommended to consider this tool as a substitute coverage survey before reaching the pre-TAS stage so that results can be interpreted and applied towards the next MDA. Using a tool such as the one presented here could alert district health staff as to where to re-direct their efforts to ensure effective drug distribution and that distributed drugs are actually ingested.

District health teams who must implement additional MDA rounds can benefit from specialized technical support based on reliable social research findings. It is recommended that national programs consider on a case-by-case basis which IUs would be helped most by a process as described here. This would ensure that valuable resources are not invested into MDA systems that continue to underperform without first having a deeper understanding of barriers to uptake and where programmatic adjustments can be made. Moreover, when additional MDA rounds are needed, staff report feeling demoralized and uncertain as to how they would secure additional funds to support further MDA rounds. Assistance with advocacy and understanding of the TAS requirements should be available to IUs that must continue MDAs beyond their planning. This is especially recommended in decentralized health systems where the local government provides some of the funding for MDA activities.

The tool and process used in this implementation research reveal that districts have the potential to implement their own feasible and affordable improvements to MDA without additional funds and with minimal technical support. It is recommended to further promote this tool and the implementation research process so that national programs can assist and guide IUs that appear to be problematic with their MDA interventions.

Furthermore a tailored approach that aims to reach specific groups in the population is an effective way to improve both drug distribution and uptake. By recognizing that all population groups will not respond to MDA in the same way, the district programs in this research reached out more efficiently and effectively to their populations and demonstrated better MDA outcomes as a result. Simple promotional materials like flowcharts, frequently asked question sheets (FAQ) and drug packaging inserts aid those individuals at the frontline of drug distribution and do not require a significant financial commitment to develop and reproduce. These tools can be tailored to the context and those factors that have been shown to influence both coverage and compliance.

The cost of improving a program’s drug coverage rates reduces the necessity for future MDA rounds. It is recommended to evaluate the cost effectiveness of this technique so that it can be balanced against the cost of additional MDA rounds where improvement in reported coverage rates does not occur.

Finally a research tool based on people’s experiences with the MDA program provided reliable and valid results that could be interpreted into feasible and applicable recommendations for the LF program. It is recommended that the use of this micronarrative methodology be more widely explored in other areas where health behavior is studied.
